# Non-communicable disease co-morbidity and associated factors in tuberculosis patients: A cross-sectional study in Gabon

**DOI:** 10.1016/j.eclinm.2022.101316

**Published:** 2022-02-27

**Authors:** BR Adegbite, JR Edoa, JBP Agbo Achimi Abdul, M Epola, C Mevyann, JC Dejon-Agobé, JF Zinsou, YJ Honkpehedji, SG Mpagama, AS Alabi, PG Kremsner, K Klipstein-Grobusch, AA Adegnika, MP Grobusch

**Affiliations:** a German Center for Infection Research, Centre de Recherches Médicales de Lambaréné and African Partner Institution, Lambaréné, Gabon; b Center of Tropical Medicine and Travel Medicine, Department of Infectious Diseases, location AMC, Amsterdam Public Health, Amsterdam Infection and Immunity, University of Amsterdam, Amsterdam University Medical Centers, location AMC, Meibergdreef 9, Amsterdam 1105 AZ, the Netherlands; c Universität Tübingen and German Center for Infection Research, Institut für Tropenmedizin, Tübingen, Germany; d Department of Parasitology, Leiden University Medical Center, Leiden, the Netherlands; e Kibong'oto Infectious Diseases Hospital - Sanya Juu Siha/Kilimanjaro Clinical Research Institute Kilimanjaro Tanzania, Mae Street, Lomakaa Road, Siha Kilimanjaro, Tanzania; f Julius Global Health, Julius Center for Health Sciences and Primary Care, University Medical Center Utrecht, Utrecht University, Utrecht, the Netherlands; g Division of Epidemiology and Biostatistics, School of Public Health, Faculty of Health Sciences, University of the Witwatersrand, Johannesburg, South Africa; h Masanga Medical Research Unit (MMRU), Masanga, Sierra Leone; i Institute of Infectious Diseases and Molecular Medicine (IDM), University of Cape Town, Cape Town, South Africa

**Keywords:** Tuberculosis, Co-morbidity, Diabetes, Hypertension, Obesity, Non-communicable diseases

## Abstract

**Background:**

There are only limited data from resource-limited settings available on the prevalence of non-communicable diseases and associated risk factors of tuberculosis patients. This study investigated non-communicable disease co-morbidity in tuberculosis patients from Moyen Ogooué Province, Gabon.

**Methods:**

All patients aged 18 years or older consulting for tuberculosis (TB) symptoms in Gabon's Moyen Ogooué province and neighbouring provinces from November 2018 to November 2020 were screened for diabetes mellitus, hypertension, and risk factors thereof (obesity, dyslipidaemia, smoking and alcohol consumption). Logistic regression was performed to identify factors associated with TB-diabetes and TB-hypertension co-morbidities.

**Findings:**

Of 583 patients included, 227 (39%) were diagnosed with tuberculosis. In tuberculosis-confirmed patients, the prevalences of hypertension and diabetes were 16·3% and 12·8%, respectively. The prevalence of diabetes was twice as high in tuberculosis patients compared to non-tuberculosis patients. Factors independently associated with hypertension-tuberculosis co-morbidity were age >55 years (aOR=8·5, 95% CI 2·43, 32·6), age 45–54 years (aOR=4.9, 95%CI 1.3–19.8), and moderate alcohol consumption (aOR=2·4; 95% CI 1·02- 5·9), respectively. For diabetes-tuberculosis co-morbidity, age >55 years was positively (aOR=9·13; 95% CI 2·4–39·15), and moderate alcohol consumption inversely associated (aOR=0·26, 95% CI 0·08- 0·73). One-hundred-and-four (46%) of the tuberculosis patients had at least either dyslipidaemia, hypertension, diabetes, or obesity with a majority of newly-diagnosed hypertension and diabetes.

**Interpretation:**

Integration of screening of non-communicable diseases and their risk factors during TB assessment for early diagnosis, treatment initiation and chronic care management for better health outcomes should be implemented in all tuberculosis healthcare facilities.

**Funding:**

This study was supported by WHO AFRO/TDR/EDCTP (2019/893,805) and Deutsches Zentrum für Infektiologie (DZIF/ TTU 02.812).

**Research in context**

**Evidence before this study**

We searched PubMed and Google Scholar for studies published prior to November 2018 assessing the integrated care for non-communicable diseases (NCDs), amongst tuberculosis patients, to identify the burden of NCDs and tuberculosis co-morbidity in Gabon and Central Africa region. Applying the search terms ‘integrated care’, ‘point-of-care’, ‘tuberculosis’, ‘non-communicable diseases’, ‘diabetes mellitus’, ‘hypertension’, ‘obesity‘ without language restrictions did not identify studies integrating point-of-care testing for both blood glucose, and cholesterol into a facility-based standard of care for tuberculosis in Gabon.

**Added value of this study**

The present study indicates high prevalence of hypertension, diabetes and NCD risk factors, and co-morbidity in adult tuberculosis patients and controls in Moyen Ogooué region, Gabon. Diabetes prevalence was about twice as high in tuberculosis patients. Overall, almost half of the tuberculosis patients had at least either dyslipidaemia, hypertension, diabetes, or obesity with the majority of them with newly-diagnosed hypertension and diabetes. Factors associated with tuberculosis-diabetes and tuberculosis-hypertension co-morbidities were age older than 55 years and alcohol consumption.

**Implications of all the available evidence**

The high prevalence of hypertension, diabetes co-morbidities and risk factors thereof in tuberculosis patients indicates a systematic screening for NCDs and NCD risk factors should be integrated in tuberculosis care. Integrated chronic disease care of tuberculosis patients would improve early diagnosis, treatment initiation and management of co-morbidities for better health outcomes.

Alt-text: Unlabelled box

## Introduction

Low- and middle-income countries (LMICs) are experiencing an increasing double burden of communicable and non-communicable diseases (NCDs), with limited capacity of the health system to address non-communicable diseases in addition to endemic communicable diseases such as tuberculosis (TB) or human immunodeficiency virus (HIV).^1^^,^^2^ Current reports show a growing evidence of links between communicable diseases and NCDs, or risk factors thereof,^3^^,^^4^ such as tobacco use, physical inactivity, unhealthy diet, the harmful use of alcohol, and cardio-metabolic risk factors such as high blood pressure, overweight/obesity, and dyslipidaemia.^5^

Models of TB/HIV co-management such as ‘two diseases, one patient’ have improved early TB diagnosis and treatment amongst people with HIV infection, and improved clinical outcomes for both diseases.^6^ This concept could also be applied to non-communicable co-morbidities and tuberculosis. Clinicians receiving patients with suspected TB will need to acknowledge that they may be dealing with multiple diseases, of which some might be beyond their own core expertise area, in a single patient. Integrated screening and management could improve early diagnosis and health outcomes for both conditions.^7^

Some studies have reported NCD screening in tuberculosis patients^7^, ^8^, ^9^; however, they focused mainly on diabetes. The burden of other frequently-occurring NCDs or risk factors in patients with tuberculosis remains under-investigated. The absence of investigation of NCD co- and multi-morbidity amongst patients with TB may impact negatively on the success of TB control programmes.^6^^,^^10^ Two-way screening and integrated service management can help with TB control programmes by improving early diagnosis, treatment, and treatment outcomes. There is inadequate evidence, so far, on the feasibility and effectiveness of the screening and integrated management of NCDs in TB-suspected patients in resource-limited settings.

Gabon is a high-burden TB country,^11^^,^^12^ with an estimated incidence of 521 TB cases per 100,000 inhabitants reported by the World Health Organization (WHO) in 2019.^13^ Around 31% of deaths in Gabon are caused by NCDs.^14^ It is expected that in the coming years, Gabon will face the challenge of dealing with a continuously high burden of communicable diseases, while also needing to address the increasing burden of NCDs.^15^

The primary objective of this study was to determine the prevalence of NCDs (diabetes mellitus, hypertension) and risk factors (obesity, dyslipidaemia and smoking) in tuberculosis patients. The secondary objective of the study was to investigate factors associated with tuberculosis, TB-diabetes and TB-hypertension co-morbidities, and to assess the feasibility of integrating screening for non-communicable diseases and their risk factors into routine tuberculosis care.

## Methods

The Strengthening Reporting of Observational Studies in Epidemiology (STROBE) guideline^16^ was applied to report this study.

### Study design and setting

A cross-sectional study was performed from November 2018 to November 2020 amongst patients consulting for tuberculosis symptoms. The study was conducted across Gabon's Moyen Ogooué province and neighbouring provinces. Gabon (population of 2.17 million in 2020) is one of three African countries, together with South Africa (58.56 million) and Lesotho (2.13 million), with a tuberculosis incidence of >500/100,000 (578/100,000).^13^ The tuberculosis mortality rate is estimated at 110 per 100,000 population.^13^ The WHO estimated that 10% of Gabon's population is at risk of death due to NCDs by 2025.^14^ The Centre de Recherches Médicales de Lambaréné (CERMEL) tuberculosis laboratory serves the Moyen-Ogooué region, with a catchment area of approximately 170.000 inhabitants, and constitutes the national reference tuberculosis laboratory. In addition, patients from different parts of the country are regularly referred for consultation to CERMEL as clinical research centre,^17^, ^18^, ^19^ with its TB laboratory having evolved into the National DR-TB reference laboratory, providing support for the diagnosis of tuberculosis, patients management, and drafting and implementation of tuberculosis guidelines.

### Data collection and study procedures

All patients consulting for tuberculosis signs and symptoms referred to CERMEL's tuberculosis laboratory were invited to participate. Patients were from all the health facilities in Moyen Ogooué region and surroundings: (1) in- and outpatient departments of the Albert Schweitzer Hospital (HAS); (2) in- and outpatient departments of Georges Rawiri Regional Hospital (CHRGR); (3) the local outpatient HIV clinic (Centre de Traitement Ambulatoire [CTA]); (4) the local outpatient TB clinic (Base d'épidémiologie [BELE]); 5) CERMEL; (6) the Centre de Santé de Bifoun; (7) Centre de Santé de Ndjolé; (8) Centre de Santé de Fougamou, and nearby primary healthcare facilities from Ngounié, Nyanga, Estuaire and Ogooué-Maritime provinces. CERMEL thus represents all ports of entry for TB patients into the local and regional healthcare system. All adults (≥18 years) with a presumable TB diagnosis who attended the CERMEL tuberculosis laboratory, or who were hospitalised in one of the Lambaréné Hospitals, were screened. Patients who were unable to provide informed consent were excluded. Once agreed to participate, a written consent was obtained from the participant, and a structured questionnaire addressing sociodemographic, smoking, alcohol consumption behaviours and clinical information was administered by the study nurses. The physical examination was performed by a research physician. All patients provided two sputum samples (one at consultation and one early next morning) as suggested by the national tuberculosis control programme and reported by Adegbite et al.^20^ All patients consulting for tuberculosis signs and symptoms were considered as presumptive TB patients. Patients with positive *Mycobacterium tuberculosis* smear microscopy, MTB RIF Xpert, culture or extra-pulmonary TB (EPTB) were considered confirmed TB. A diagnosis of extra-pulmonary confirmed TB was based on positive MTB RIF Xpert/culture, or ultrasound scanning and clinical evidence consistent with active EPTB in the absence of an alternative diagnosis, and the decision of the clinician to treat with a full course of TB chemotherapy.

Fasting capillary blood glucose was determined using rapid blood glucose (RBG) metre strips (ACON, San Diego, CA, USA). A twelve-hours overnight fasting blood sample was collected from all patients before anti-tuberculosis treatment was initiated, to measure glucose, glycosylated haemoglobin (HbA1c), triglycerides (TG), high-density lipoprotein cholesterol (HDL‐C), and total cholesterol (TC). Diagnosis of diabetes was based on previous medical history of diabetes in the medical file of the patient or on WHO criteria^21^ for the classification of glucose tolerance (diabetic: fasting glucose ≥7·0 mmol/L, the HbA1c level (≥6·5%), or random glucose ≥11·1 mmol/L with clinical symptoms). Participants without medical history of diabetes but with abnormal RBG and normal HbA1c were tested a second time (a week later) assessing plasma glucose to confirm the diagnosis of diabetes mellitus. Blood pressure was measured by a nurse after the subject had rested for at least five minutes, using an automatic digital blood pressure monitor (Spengler, Aix-en-Provence, France). For each patient, two readings were recorded (left and right arm each once).^22^^,^^23^ Arterial hypertension was defined as diastolic blood pressure ≥90 mm of Hg and/or a systolic blood pressure ≥ 140 mm Hg, and/or use of any participant-reported antihypertensive drug. Participants without medical history of hypertension (HT) with uncontrolled blood pressure or high blood glycaemia on screening were invited to come to the clinic two weeks later for confirmation. If the diagnosis was indeed confirmed, they were referred for specialist treatment. Dyslipidaemia was defined by the presence of high total cholesterol (TC ≥240 mg/dL), low-density lipoprotein cholesterol (LDL-C ≥ 160 mg/dL), high triglyceride (TG ≥150 mg/dL), and low high-density lipoprotein cholesterol (HDL-C < 40 mg/dL).^24^ The low-LDL-C level was calculated using the Friedewald formula (LDL=TC—HDL-1/5(TG)).^25^ All biochemical assessments were performed using Cobas® (Roche, Switzerland) clinical chemistry analysers.

Body weight was recorded in kilograms (kg) using an automated scale (Omron Healthcare, Hoofddorp, Netherlands). Height was measured in centimetres using a fixed stadiometer (SECA, Hamburg Germany). Waist circumference was measured in standing position, the inelastic tape placed around the patient hipbones, keeping the tape snugly around the waist. Body mass index (BMI) was calculated as weight (kg) divided by height (m^2^) according to the WHO international classification (18·5 to 24·9: normal; 25 to 29·9: overweight; above 30: obesity).^26^ Abdominal obesity was categorised as waist circumference (WC) ≥ 90 cm (men) and ≥80 cm (women).^27^ Participant were asked to provide an estimation of the quantity, the type and the rate of alcohol consumption. Harmful alcohol consumption was defined according to the Alcohol Use Disorders Identification Test (AUDIT) score.^28^ The AUDIT is a 10-item screening tool developed by the World Health Organization to assess alcohol use, drinking behaviour, and related problems. The possible answers to each question are scored 0, 1, 2, 3 or 4. The range of possible scores is 0 to 40, where 0 indicates a teetotaller who has never had a problem with alcohol. A value of 1 to 7 indicates a low-risk consumption according to the guidelines of the World Health Organization. Values of 8 to 15 indicate moderate alcohol use disorder.^28^ For the purpose of this study we considered only participants with an AUDIT score of 8 and higher as alcohol users. Any past medical history including asthma was collected from medical files, or self-reported by the patients.

### Sample size

All TB patients consulting in the study site during the study period were invited to be included in the study. The minimum sample size were 162 tuberculosis patients to be included. The sample size was calculated using Epi StaCalc software,^29^ based on an expected diabetes prevalence of 12%^30^ in tuberculosis patients, at a 95% confidence level and 5% precision.

### Statistical analysis

Statistical analyses were performed using RStudio (R Foundation for Statistical Computing, Vienna, Austria) software.^31^ The numeric variables were described using the median and the inter-quartile range. The proportion of diabetes, hypertension, obesity, smoking in tuberculosis and non-tuberculosis patients were determined and compared using the Chi square test. Factors associated with hypertension-tuberculosis and diabetes-tuberculosis were investigated using multivariable logistic regression. The multivariable logistic regression model was built by including clinically relevant variables such as sex, age, education, smoking, dyslipidaemia and alcohol, and factors associated with each event (hypertension and diabetes) in univariable analyses, with inclusion criteria of *p*<0·2 added. Adjusted odds ratios (aORs) with 95% confidence intervals (CIs) were reported. Feasibility and effectiveness of the screening approach were measured using the mean cost (in FCFA, USD) of the laboratory test for NCDs diagnosis, the availability of the screening tool for routine care, the number of patients needed to screen (NNS) to get one additional new DM or HT case, and the additional yield of new cases. The NNS was calculated using the Rembold formula.^32^ Additional yield of new cases of NCDs was calculated using the formula: (newly diagnosed cases × 100)/(known cases+ newly diagnosed cases).^30^

### Ethics approval and consent to participate

The study protocol was endorsed by CERMEL's institutional Scientific Review Board (SRC) and approved by the CERMEL's Institutional Ethics Committee (CEI-018/2018). Written informed consent was obtained from all participants included. The study was conducted in line with the Good Clinical Practice principles of the International Conference on Harmonization and the Declaration of Helsinki.

### Role of the funding source

The supporting funder of the study had no role in study design, data collection, data analysis, data interpretation, or writing of the report. The corresponding author, Martin P. Grobusch accessed the dataset, and the decision to submit for publication was jointly taken by all authors.

## Results

### General characteristics

**Figure 1:**
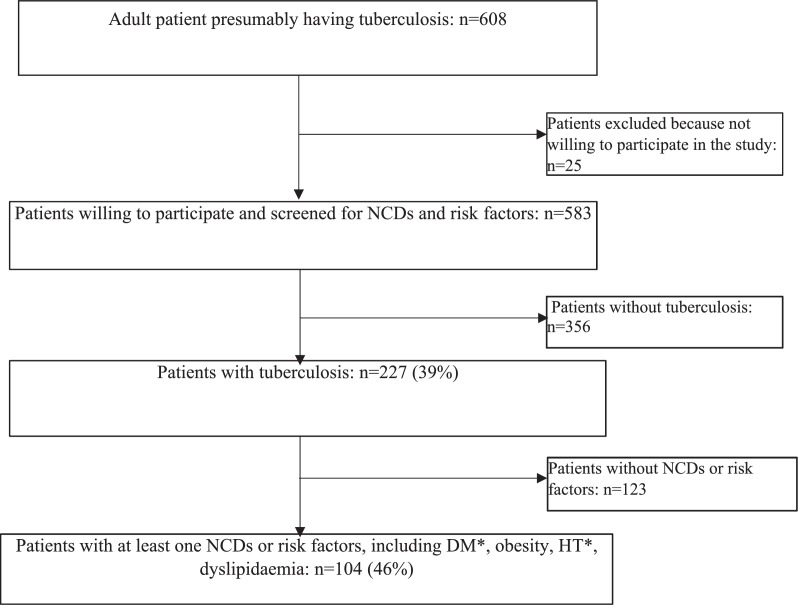
Flow of participants through the screening process. Legend *DM: diabetes mellitus, HT: hypertension, NCDs: Non-communicable diseases. All patients consulting for tuberculosis signs and symptoms were considered as presumptive TB patients. Those with laboratory-confirmed TB (or clinical/radiological strong evidence (for extra pulmonary TB) were considered as patients with tuberculosis. Obesity: Body mass index ≥30, or waist circumference (WC) ≥ 90 cm (men) and ≥80 cm (women). Dyslipidaemia: total cholesterol (TC) ≥240 mg/dL, low-density lipoprotein cholesterol (LDL-C) ≥160 mg/dL, triglyceride (TG) levels ≥150 mg/dL, or high-density lipoprotein cholesterol (HDL‐C) < 40 mg/dL. Arterial hypertension:diastolic blood pressure ≥90 mm of Hg and/or a systolic blood pressure ≥ 140 mm Hg, and/or use of any participant-reported antihypertensive drug. Diabetes: medical history of diabetes mellitus or fasting glucose ≥7.0 mmol/L, HbA1c level ≥6.5%, or random glucose ≥11.1 mmol/L with clinical symptoms.

Table 1 summarises the study population characteristics. A total of 583 patients presumably having TB were included in the study, of which 322 (55.2%) were male; the median age was 39 [IQR 29–51] years; and 144 (24·7%) were HIV-positive, amongst whom 49 (34%) were taking HIV antiretroviral combination therapy. A total of 208 (35·7) were underweight. The overall prevalences of hypertension and diabetes were 22·8(133) and 8·7 (51), respectively.Thirty seven (37/51; 73%) of the diabetes patients knew their status. Amongst them only 27%(10/37) had normal glucose levels. A total of 227 (39%) patients were diagnosed with tuberculosis (Figure 1), of which 8 (3·5%) had extrapulmonary tuberculosis, and 14 (6·2%) had multidrug-resistant tuberculosis.

**Table 1:** Sociodemographic characteristics of TB and non-TB patients (total=583).

Characteristics	All	No Tuberculosis	Tuberculosis
	*N* *=* *583(%)*	*N = 356 (61%)*	*N = 227 (39%)*
**Age group**			
18–34 years	195 (33·4)	104 (29·2)	91 (40·1)
35–44 years	181 (31·0)	101 (28·4)	80 (35·2)
45–54 years	81 (13·9)	58 (16·3)	23 (10·1)
≥ 55 years	126 (21·6)	93 (26·1)	33 (14·5)
**Sex**			
F	261 (44·8)	157 (44·1)	104 (45·8)
M	322 (55·2)	199 (55·9)	123 (54·2)
**Area of residence**			
Rural	166 (28·5)	93 (26·1)	73 (32·2)
Urban	417 (71·5)	263 (73·9)	154 (67·8)
**Educational attainment**			
None	54 (9·3)	40 (11·2)	14 (6·2)
Primary	150 (25·7)	99 (27·8)	51 (22·5)
Secondary	344 (59·0)	196 (55·1)	148 (65·2)
University	35 (6·00)	21 (5·9)	14 (6·2)
**Incomes**			
Monthly fixed	140 (24·0)	96 (27·0)	44 (19·4)
Daily fixed	44 (7·6)	30 (8·4)	14 (6·2)
Occasional	82 (14·1)	57 (16·0)	25 (11·0)
No income	317 (54·4)	173 (48·6)	144 (63·4)
Size of household Median [IQR]	5 [3–7]	4 [2–7]	5 [3–7]

IQR: interquartile range.

### Prevalence of diabetes, hypertension, and NCD risk factors in tuberculosis patients

Twenty-nine (17 with history of diabetes treatment, nine with repeated fasting glucose ≥7·0 mmol/L, and three with HbA1c≥6·5%) TB patients had diabetes; the prevalence of diabetes was 12·8% (29 /227). Hypertension prevalence in tuberculosis-confirmed patients was 16·3% (37/227), the prevalence of dyslipidaemia was 17·6% (40/227), and the prevalence of obesity (including abdominal obesity or BMI above 30) was 6·2% (14/227) Table 2. summarises the prevalence of diabetes, hypertension, obesity, dyslipidaemia in all of participants, in patient with confirmed tuberculosis and those without tuberculosis.

**Table 2:** Clinical characteristics in TB and non-TB patients (total=583).

Characteristic	All	No Tuberculosis	Tuberculosis
	*N* *=* *583(%)*	*N = 356 (61%)*	*N = 227 (39%)*
**HIV infection**			
Negative	439 (75·3)	291 (81·7)	148 (65·2)
Positive	144 (24·7)	65 (18·3)	79 (34·8)
**Medical History of tuberculosis**			
No	514 (88·2)	317 (89·0)	197 (86·8)
Yes	69 (11·8)	39 (11·0)	30 (13·2)
**Diabetes status**			
No-diabetes	532 (91·3)	334 (93·8)	198 (87·2)
Diabetes	51 (8·7)	22 (6·18)	29 (12·8)
**Medical History of diabetes**			
No	546 (93·7)	342 (95)	204 (83)
Yes	37 (6·3)	20 (5)	17 (7)
**Hypertension**			
No	450 (77·2)	260 (73·0)	190 (83·7)
Yes	133 (22·8)	96 (27·0)	37 (16·3)
**Medical history of hypertension**			
No	525 (90)	310 (86·1)	215(94·7)
Yes	58 (10)	46 (12·9)	12 (5·3)
**Smoking**			
No	365 (62·6)	219 (61·5)	146 (64·4)
Yes currently	41 (7·0)	25 (7·0)	16 (7·0)
Yes, but I quit smoking	177 (30·4)	112 (31·5)	65 (28·6)
**Alcohol consumption**			
No	358 (61·4)	222 (62·4)	136 (59·9)
Yes	225 (38·6)	134 (37·6)	91 (40·1)
**Dyslipidaemia**			
No	496 (85·1)	307 (86·2)	189 (83·3)
Yes	87 (14·9)	49 (13·8)	38 (16·7)
**Body Mass Index**			
Normal BMI	282 (48·4)	176 (49·4)	106 (46·7)
Underweight	208 (35·7)	105 (29·5)	103 (45·4)
Overweight	48 (8·2)	37 (10·4)	11 (4·9)
Obese	45 (7·7)	38 (10·7)	7 (3·1)
**Abdominal obesity**			
No	532 (91·3)	314 (88·2)	218 (96·0)
Yes	51 (8·8)	42 (11·8)	9 (4)

Body mass index less to 18·5 underweight,18·5 to <25, normal; 25·0 to <30, overweight; ≥30, obesity. Abdominal obesity: waist circumference (WC) ≥ 90 cm (men) and ≥80 cm (women). Dyslipidaemia: the presence total cholesterol (TC) ≥240 mg/dL, low-density lipoprotein cholesterol (LDL-C) ≥160 mg/dL, triglyceride (TG) levels ≥150 mg/dL, or high-density lipoprotein cholesterol (HDL‐C) < 40 mg/dL, Alcohol consumption(moderate alcohol use disorder): audit score of 8 or more; Arterial hypertension:s diastolic blood pressure ≥90 mm of Hg and/or a systolic blood pressure ≥ 140 mm Hg, and/or use of any participant-reported antihypertensive drug; Diabetes: medical history of diabetes mellitus or fasting glucose ≥7.0 mmol/L, the HbA1c level (≥6.5%), or random glucose ≥11.1 mmol/L with clinical symptoms.

### Prevalence of TB and NCDS risk factors or HIV co-morbidity (occurrence of one or more medical conditions)

**Figure 2:**
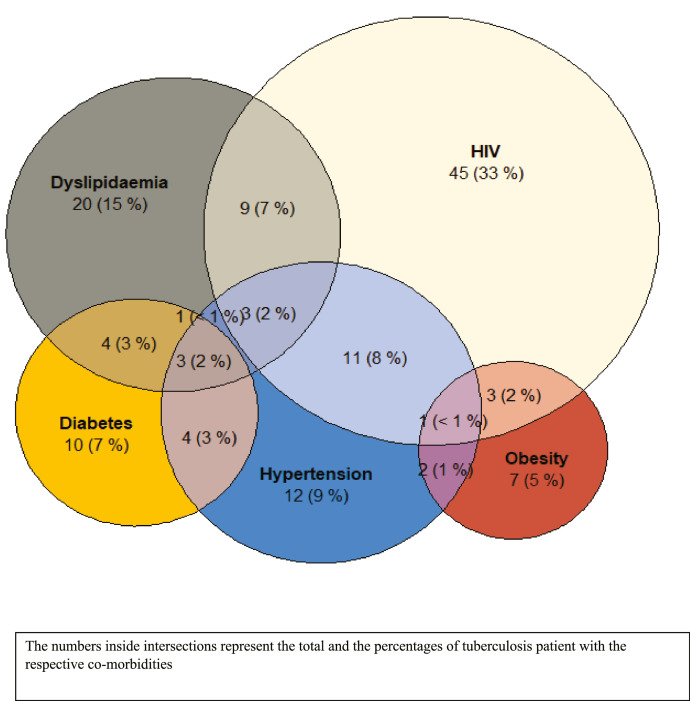
Venn-Euler diagram representing the distribution of participants according to their co-morbidities or NCDs risk factors. The numbers inside intersections represent the total and the percentages of tuberculosis patient with the respective co-morbidities.

A total of 46% (104/227) of tuberculosis patients had at least one co-morbidity or NCD risk factor (including, hypertension, diabetes, dyslipidaemia, obesity).When taking into account HIV, this prevalence is 65%(147/227). The most-common combination of co-morbidity or NCDs risk factors in TB patients was HIV-hypertension and HIV-dyslipidaemia (Figure 2).

### Factors associated with tuberculosis

Compared to patients 55 years or older, the patients within the age categories of 18–34 years and 35–45 years were more likely to present with tuberculosis (aOR=2·02; 95% CI 1·11–3·75, and aOR=2·09; 95% CI 1·16–3·83, respectively). Other factors associated with tuberculosis in multivariable analysis were diabetes (aOR=4·24; 95% CI 2·17–8·53), HIV infection (aOR=2·31; 95% CI 1·49–3·58), rural residency (aOR=1·54; 95% CI 1·01–2·35) and obesity (aOR=0·28; 95% CI 0.11–0.65), Table 3.

**Table 3:** Univariable and multivariable risks factor associated with tuberculosis.

Dependant: tuberculosis		Univariable or (95%ci, p· value)	Adjusted or (95%ci, p· value)
Age group	≥ 55 years	Ref	Ref
	45–54 years	1·12 (0·59–2·08, *p* = 0·73)	1·15 (0·57–2·31, *p* = 0·69)
	35–44 years	2·23 (1·37–3·69, *p* = 0·01)	2·09 (1·16–3·83, *p* = 0·02)
	18–34 years	2·47 (1·53–4·05, *p*<0·001)	2·02 (1·11–3·75, *p* = 0·02)
Sex	F	Ref	Ref
	M	0·93 (0·67–1·30, *p* = 0·68)	1·06 (0·70–1·63, *p* = 0·77)
Area of living	Urban	Ref	Ref
	Rural	1·34 (0·93–1·93, *p* = 0·12)	1·54 (1·01–2·35, *p* = 0·04)
Education attainment	None	Ref	Ref
	Primary	1·47 (0·75–3·03, *p* = 0·28)	1·05 (0·48–2·34, *p* = 0·90)
	Secondary	2·16 (1·16–4·24, *p* = 0·02)	1·41 (0·67–3·06, *p* = 0·37)
	University	1·90 (0·77–4·78, *p* = 0·16)	1·42 (0·50–4·08, *p* = 0·51)
Income	Monthly fixed income	Ref	Ref
	Daily fixed income	1·02 (0·48–2·08, *p* = 0·96)	0·74 (0·32–1·67, *p* = 0·48)
	Occasional income	0·96 (0·53–1·72, *p* = 0·88)	0·86 (0·43–1·68, *p* = 0·65)
	No income	1·82 (1·20–2·78, *p* = 0·01)	1·57 (0·97–2·58, *p* = 0·07)
Diabetes Status	No	Ref	Ref
	Yes	2·22 (1·25–4·02, *p* = 0·01)	4·24 (2·17–8·53, *p*<0·001)
Hypertension	No	Ref	Ref
	Yes	0·53 (0·34–0·80, *p* = 0·01	0·78 (0·48–1·27, *p* = 0·32)
Smoking	No	Ref	Ref
	Yes currently	0·96 (0·49–1·84, *p* = 0·9)	0·93 (0·42–2·01, *p* = 0·86)
	Yes, but I quit smoking	0·87 (0·60–1·26, *p* = 0·46)	0·79 (0·50–1·24, *p* = 0·30)
Alcohol consumption	No	Ref	Ref
	Yes	1·11 (0·79–1·56, *p* = 0·55)	1·30 (0·88–1·94, *p* = 0·19)
Body Mass Index	Normal	Ref	Ref
	Obese	0·31 (0·12–0·67, *p* = 0·01)	0·28 (0·11–0·65, *p* = 0·001)
	Overweight	0·49 (0·23–0·98, *p* = 0·05)	0·53 (0·23–1·12, *p* = 0·11)
	Underweight	1·63 (1·13–2·35, *p* = 0·01)	1·43 (0·96–2·12, *p* = 0·08)
Dyslipidaemia	No	Ref	Ref
	Yes	1·28 (0·81–2·01, *p* = 0·27)	1·21 (0·73–1·99, *p* = 0·45)
HIV infection	Negative	Ref	Ref
	Positive	2·39 (1·63–3·51, *p*<0·001)	2·31 (1·49–3·58, *p*<0·001)

Ref = Reference parameter; Body mass index less to 18·5 underweight,18·5 to <25, normal; 25·0 to <30, overweight; ≥30, obesity. Dyslipidaemia:the presence total cholesterol (TC) ≥240 mg/dL, low-density lipoprotein cholesterol (LDL-C) ≥160 mg/dL, triglyceride (TG) levels ≥150 mg/dL, or high-density lipoprotein cholesterol (HDL‐C) < 40 mg/Dl; Alcohol consumption(moderate alcohol use disorder):audit score of 8 or more; Arterial hypertension:diastolic blood pressure ≥90 mm of Hg and/or a systolic blood pressure ≥ 140 mm Hg, and/or use of any participant-reported antihypertensive drug; Diabetes: medical history of diabetes or fasting glucose ≥7.0 mmol/L, the HbA1c level (≥6.5%), or random glucose ≥11.1 mmol/L with clinical symptoms; OR: odds ratio, aOR: adjusted odds ratio.

### Factor associated with diabetes, and HBP in tuberculosis patients

Age older than 55 years (aOR=6·99; 95%CI 2·10–25·44) and moderate alcohol consumption (aOR=0·27; 95% CI 0.09–0.75) were independently associated with diabetes in multivariable analysis (Table 4). Age older than 55 years (aOR=7·48; 95% CI 2·36–25·80) and 45–54 years (aOR=4·35; 95% CI 1·26–15·49); a family history of diabetes (aOR=3·05; 95% CI 1·08–8·38), moderate alcohol consumption (aOR=2·38; 95% CI 1·01- 5·7) were positively and being underweight (aOR=0·39; 95% CI (0·15–0·94) inversely associated with hypertension (Table 5).

**Table 4:** Factors associated with diabetes in tuberculosis patients (29 diabetes cases / in 229 TB cases).

		Univariable OR(95%CI, P· Value)	Adjusted OR(95%CI, P· Value)
Age group	18–34 years	Ref	Ref
	35–44 years	1·33 (0·46–3·97, *p* = 0·59)	1·39 (0·43–4·63, *p* = 0·58)
	45–54 years	1·14 (0·16–5·15, *p* = 0·87)	0·47 (0·05–2·80, *p* = 0·44)
	≥ 55 years	6·86 (2·46–20·52, *p*<0·001)	6·99 (2·10–25·44, *p* = 0·002)
Sex	F	Ref	Ref
	M	2·47 (1·08–6·18, *p* = 0·04)	1·72 (0·57–5·40, *p* = 0·34)
Income	Monthly fixed income	Ref	Ref
	Daily fixed income	1·80 (0·41–7·07, *p* = 0·40)	3·72 (0·67–20·50, *p* = 0·12)
	Occasional income	0·86 (0·21–3·08, *p* = 0·81)	0·66 (0·12–3·20, *p* = 0·61)
	No income	0·45 (0·17–1·20, *p* = 0·09)	0·54 (0·18–1·75, *p* = 0·28)
Hypertension	No	Ref	Ref
	Yes	1·78 (0·66–4·37, *p* = 0·22)	1·48 (0·40–4·96, *p* = 0·53)
Smoking	No	Ref	Ref
	Yes currently	1·25 (0·18–5·07, *p* = 0·78)	1·06 (0·12–6·38, *p* = 0·95)
	Yes, but I quit smoking	1·98 (0·85–4·50, *p* = 0·10)	1·47 (0·51–4·13, *p* = 0·46)
Alcohol consumption	No	Ref	Ref
	Yes	0·53 (0·21–1·21, *p* = 0·14)	0·27 (0·09–0·75, *p* = 0·02)
Body Mass Index	Normal	Ref	Ref
	Obese	1·01 (0·05–6·52, *p* = 0·99)	0·97 (0·04–9·40, *p* = 0·97)
	Overweight	1·35 (0·19–5·90, *p* = 0·71)	1·82 (0·19–11·53, *p* = 0·55)
	Underweight	0·73 (0·31–1·65, *p* = 0·44)	0·99 (0·37–2·66, *p* = 0·98)
Family medical history of diabetes	No	Ref	Ref
	Yes	2·20 (0·80–5·49, *p* = 0·10)	2·79 (0·81–8·93, *p* = 0·08)
Dyslipidaemia	No	Ref	Ref
	Yes	0·50 (0·12–1·53, *p* = 0·27)	0·63 (0·13–2·25, *p* = 0·51)
HIV infection	Negative	Ref	Ref
	Positive	0·56 (0·21–1·31, *p* = 0·20)	0·58 (0·19–1·60, *p* = 0·31)

Ref = Reference parameter, Body mass index less to 18·5 underweight,18·5 to <25, normal; 25·0 to <30, overweight; ≥30, obesity. Dyslipidaemia:the presence total cholesterol (TC) ≥240 mg/dL, low-density lipoprotein cholesterol (LDL-C) ≥160 mg/dL, triglyceride (TG) levels ≥150 mg/dL, or high-density lipoprotein cholesterol (HDL‐C) < 40 mg/dL, Alcohol consumption(moderate alcohol use disorder):audit score of 8 or more; Arterial hypertension: diastolic blood pressure ≥90 mm of Hg and/or a systolic blood pressure ≥ 140 mm Hg, and/or use of any participant-reported antihypertensive drug; Diabetes: medical history of diabetes or fasting glucose ≥7.0 mmol/L, the HbA1c level (≥6.5%), or random glucose ≥11.1 mmol/L with clinical symptoms; OR: odds ratio, aOR: adjusted odds ratio.

**Table 5:** Factors associated with hypertension in tuberculosis patients (37 cases of hypertension/227 TB patients).

		Univariable OR (95%CI, P· Value)	Adjusted OR(95%CI, P· Value)
Age group	18–34 years	Ref	Ref
	35–44 years	1·52 (0·54–4·45, *p* = 0·428)	1·21 (0·40–3·77, *p* = 0·74)
	45–54 years	6·40 (2·02–20·95, *p* = 0·002)	4·35 (1·26–15·49, *p* = 0·02)
	≥ 55 years	7·80 (2·83–23·23, *p*<0·001)	7·48 (2·36–25·80, *p* = 0·01)
Sex	F	Ref	Ref
	M	0·87 (0·43–1·78, *p* = 0·70)	0·48 (0·18–1·23, *p* = 0·13)
Diabetes	No	Ref	Ref
	Yes	1·78 (0·66–4·37, *p* = 0·23)	1·18 (0·34–3·72, *p* = 0·78)
Smoking	No	Ref	Ref
	Yes currently	1·88 (0·49–5·97, *p* = 0·31)	1·20 (0·27–4·81, *p* = 0·79)
	Yes, but I quit smoking	1·15 (0·50–2·49, *p* = 0·73)	0·93 (0·32–2·57, *p* = 0·88)
Alcohol consumption	No	Ref	Ref
	Yes	2·25 (1·11–4·66, *p* = 0·02)	2·38 (1·01–5·73, *p* = 0·04)
Body Mass Index	Normal	Ref	Ref
	Obese	0·60 (0·03–3·77, *p* = 0·64)	0·73 (0·03–5·95, *p* = 0·79)
	Overweight	0·80 (0·12–3·39, *p* = 0·78)	0·68 (0·08–3·81, *p* = 0·69)
	Underweight	0·43 (0·19–0·92, *p* = 0·03)	0·39 (0·15–0·94, *p* = 0·04)
Family medical history of diabetes	No	Ref	Ref
	Yes	2·83 (1·17–6·52, *p* = 0·01)	3·05 (1·08–8·38, *p* = 0·03)
Dyslipidaemia	No	Ref	Ref
	Yes	0·69 (0·22–1·77, *p* = 0·47)	0·78 (0·23–2·27, *p* = 0·66)
HIV infection	Negative	Ref	Ref
	Positive	1·34 (0·64–2·75, *p* = 0·42)	1·37 (0·58–3·22, *p* = 0·46)

Ref = Reference parameter, Body mass index less to 18·5 underweight,18·5 to <25, normal; 25·0 to <30, overweight; ≥30, obesity. Dyslipidaemia:the presence total cholesterol (TC) ≥240 mg/dL, low-density lipoprotein cholesterol (LDL-C) ≥160 mg/dL, triglyceride (TG) levels ≥150 mg/dL, or high-density lipoprotein cholesterol (HDL‐C) < 40 mg/dL, Alcohol consumption(moderate alcohol use disorder):audit score of 8 or more; Arterial hypertension: diastolic blood pressure ≥90 mm of Hg and/or a systolic blood pressure ≥ 140 mm Hg, and/or use of any participant-reported antihypertensive drug; Diabetes: medical history of diabetes or fasting glucose ≥7.0 mmol/L, the HbA1c level (≥6.5%), or random glucose ≥11.1 mmol/L with clinical symptoms; OR: odds ratio, aOR: adjusted odds ratio.

### Additional yield of diabetes mellitus, HBP and feasibility of systematic screening of diabetes and NCDs factors in TB patients

In TB patients, the additional yield of diabetes case screening was 41% (12/29); the NNS to detect one new case of diabetes was 13. The additional yield of hypertension cases on screening was 68% (25/37), and the NNS to detect one new case of hypertension was 8.

The feasibility of integrating the routine screening of NCD in TB patients was assessed by using two tools: The cost of NCDs screening and the point-of care availability of a screening tool. The average cost for a patient without medical insurance is 43,750 FCFA (78·12 USD). Those covered by the medical insurance would pay 13,675 FCFA (24·42 USD). A total of 86% (486/583) of presumptive TB patients had a medical insurance and 83% (189/227) of tuberculosis patients were insured. Therefore, screening of NCDs will not significantly induce additional cost for the patients.

## Discussion

We found the prevalence of NCDs; DM and hypertension amongst TB patients were 13% and 16%, respectively. The prevalence of DM amongst TB patients in our study is similar to reports from Nigeria (12%),^33^ and higher than what was reported from Ethiopia (8·3%)^34^ and Uganda (8·5%), respectively. The differences in prevalence across studies might be explained by the diabetes burden in the general population of each country. The differences in dietary habits, behaviours, and the methods of DM screening might explain the variation in prevalence of DM amongst TB patients in other studies as compared to ours. The proportion of new diabetes cases in TB patients (5·3%,12/227) identified in our study is similar to that reported from Ethiopia (4.9%). The high proportion of newly-diagnosed diabetes cases in our study highlights the magnitude of the problem, low awareness, and the importance to systematically screen for DM, HT and NCD risk factors in general amongst TB patients.^7^ As reported previously, in our study, diabetes in tuberculosis patients is associated with older age.^35^ Our findings suggest that tuberculosis patients who consumed (moderately, or more) alcohol had lower odds (aOR=0·26; 95% CI (0·08–0·73) to present with diabetes. Data on the association between alcohol consumption and the risk of diabetes are controversial.^36^ Several meta-analyses suggest that light and moderate alcohol consumption are associated with a lower risk of diabetes.^36^, ^37^, ^38^, ^39^ As many observational studies, alcohol consumption in our study is based on self-reporting; and the quantities consumed by the patients could not be verified with accuracy; therefore, the findings should be interpreted with caution. In the context of this study, we were mainly interested in the smoking/harmful alcohol use status of the patients. Our previous study in the same study population reported the burden of smoking and alcohol use in TB patients by reporting the quantity smoked or drunk per day.^20^

Few studies in LMICs investigated the burden of hypertension in tuberculosis patients. The prevalence of hypertension in TB patients in the present study is similar to the 19% reported by Segafredo et al. from Angola.^7^ Older age and alcohol consumption are associated with hypertension in tuberculosis patients in our study; a finding in line with previous reports.^35^^,^^40^ As reported by other studies,^35^^,^^41^^,^^42^ a considerable proportion of our study population did not know their diabetes and hypertension status. The additional case yield from TB patient screening was 41% (12/29) and 68% (25/37) for diabetes and hypertension, respectively. Furthermore, 46% (104/227) of tuberculosis patients had at least one comorbidity (hypertension, diabetes) or NCD risk factor (dyslipidaemia, obesity). A study in Indonesia reported 35·5% of patients with co-morbidities (asthma, diabetes, hypertension, myocardial infarction, kidney disease, neoplasia) in tuberculosis patients.^41^ Another study conducted in the Philippines reported 40% of subjects with co-morbidities amongst tuberculosis patients.^43^ On the one hand, the slight difference in theses proportions compared to our study might be due to the co-morbidity assessment methodology in each study. For example, the study from the Philippines focused on diabetes, severe anaemia, obesity, and under-nutrition. On the other hand, the difference could be explained by the NCDs respective epidemiological peculiarities of the country or continent where the studies have been conducted. We were not able to find any study from sub-Saharan Africa that reported the prevalence of at least two NCDs, or co-morbidities risk factors, in tuberculosis patients. An un-diagnosed co-morbidity in tuberculosis patients might worsen tuberculosis outcome, and impact negatively on the sucess of a TB control programme.^6^ Our findings stress the utility and feasability of routine screening for diabetes and other non-communicable disease in all patients visiting a health care facility irrespective of the primary motif of consultation. Lessons learned from operational research aiming to integrate chronic care using the vertical HIV programmes as starting point is that those should be implemented for tuberculosis, too.^44^ The tests used in our study are widely available and could serve a useful screening function. Given the numbers needed to test to detect a new case for each of the non-communicable diseases, it seems feasible to incorporate routine screening and secondary prevention of common NCDs. Furthermore, the vast majority of tuberculosis patients in our study has medical insurance. Therefore, screening for NCDs would not be expensive for patients. However, systematic screening for non-communicable diseases during TB care would require capacity building and a more inclusive focus on the patient's general health and well-being. In our study, we worked with nurses who are in charge of tuberculosis patients screening in routine activities. Additional screening of NCDs in tuberculosis patients did not seem to be a challenge. All TB care centres in Gabon have the diagnostic tools used in our study at hand (except for HbAc1 measurement); this diabetes point-of-care rapid capillary test could be provided. The nurses and physicians could be trained continuously in the field to provide screening of NCDs. The national tuberculosis control program in many LMICs integrated successfully the screening of HIV in tuberculosis patients. This could be easily extended to NCDs. It will enhance ‘patient-centred care’, in line with the World Health Organization's End TB strategy.^45^

The strengths of our study were the enrolment of a large number of participants in a consecutive manner for 24 months in order to cover seasonality factors that might affect the incidence of tuberculosis cases. The study was integrated in routine TB activities to safeguard representativeness of the participants included. Only 25 patients declined to participate in the study. The fact that patients from the whole region towards particular social strata were captured without bias, that way limiting the risk of selection bias. Furthermore, the screening of NCDs in our study was not limited to confirmed TB only, but extended to all patients coming with TB symptoms to make sure that all patients were given an opportunity of earlier diagnosis of our target NCD conditions. To our knowledge, our study is the first performing the screening for NCDs in TB-presumptive patients and assessing the burden of NCDs and TB comorbidities in the central African region. All of the resources needed (laboratory reagents and machines; qualified medical staff) for the screening are available in all of the TB clinics referring patients to the CERMEL TB laboratory. There was no particular challenge regarding our staff collecting data on additional clinical information related to NCDs and risk factor during the screening of TB patients. Our study showed that the screening of diabetes and other co-morbidities is feasible in TB health care facilities. Moreover, a higher proportion of patients with national medical insurance coverage in Gabon provides additional evidence of the feasibility of the systematic screening without higher additional cost for the patients. This might not be the case in other sub-Saharan countries; however, systematic screening using at least diabetes point-of-care rapid capillary testing could be feasible.There are some limitations of our study. Due to self-reporting of some behaviour like alcohol drinking and smoking, social desirability bias may have affected the study findings. The physical or sportive activities of our study population as well as neoplasia co-morbidity, and kidney diseases were not reported. The same applies to chronic obstructive lung disease (COPD) as pulmonary function tests are still not available on site. The lipid profile is known to be affected by acute infections^46^ and antiretroviral treatment. The antiretroviral treatment data were not collected in our study. In the present study, HbAc1 measurement led to the diagnosis of three additional (3/29,10%) TB patients with diabetes. In patients without medical history of diabetes, the concordance between fasting glucose test and HbA1C was 75%(9/12). Gupte et al. reported in their study^47^ that the HbA1c levels declined during anti-tuberculosis treatment, suggesting that repeating HbA1c testing at treatment completion could reduce the risk of manifest diabetes. On the other hand, a scoping review on the use of HbA1c in the African setting suggested caution when interpreting results, since some co-morbidities such as anaemia and HIV infection could affect HbA1c levels.^48^ Due to the cross-sectional design of the present study, we were not able to repeat HbA1c measurement at the end of the treatment period. However, the fasting glucose tests were repeated at the beginning in patients without medical history of diabetes (presenting with or without hyperglycaemia), to reduce the risk of diabetes misdiagnosis. However, the interpretation of our findings should be conducted with caution. We did not collect qualitative information about the acceptability of the study by a representative medical staff in charge of TB in the region, so the interpretation of the feasibility data should be done with caution. However, our study provided valuable epidemiological data on DM and NCDs in Gabon's Moyen-Ogooué region and suggest the feasibility of integrating systematic screening of DM and NCDs condition during TB consultation. Qualitative and quantitative studies investigating the feasibility, the cost and effectiveness in the national level should be performed to adjust appropriate public health strategy.

## Declaration of interests

None of the authors have a competing interest to declare.

## Supplementary materials

Image, application 1
